# Illinois State Medical Society

**Published:** 1856-04

**Authors:** 


*Illinois State Medical Society.*

We would remind our readers that the next regular annual meeting of this State Society, is to be held in Vandalia, commencing on the first Tuesday in June. The Delegates from that place, who attended the last annual meeting in Bloomington, assured the Society that a meeting in Vandalia would call out a full delegation from the Profession in the Southern part of the State. We hope it will be so, and also that the profession in the Northern and middle parts of the State will find in this hope an additional motive for the selection of such delegates as will not fail to attend the meeting. We copy from the Transactions a list of the Committees from which reports may be expected at the next meeting, and also the names of the Delegates to the American Medical Association.

